# Laser‐Induced Coal‐Based Porous Graphene as Anode Toward Advanced Lithium‐Ion Battery

**DOI:** 10.1002/advs.202504592

**Published:** 2025-05-08

**Authors:** Xiao Ma, Shiyue Li, Wenhao Tang, Ruiping Liu, Zilong Fu, Shaoqing Wang

**Affiliations:** ^1^ School of Geoscience and Surveying Engineering China University of Mining & Technology (Beijing) Beijing 100083 P. R. China; ^2^ School of Chemical and Environmental Engineering China University of Mining & Technology (Beijing) Beijing 100083 P. R. China

**Keywords:** anode, coal, laser‐induced, lithium‐ion battery, porous graphene

## Abstract

Graphene have been considered as the one of the most promising anode materials for the next generation lithium‐ion batteries (LIBs) due to its unique properties compared to that of the commercial available graphite anode. However, the tedious preparation process, high cost and easy aggregation of 2D graphene caused by the strong van der Waals interactions among nanosheets affect the high reversible capacity of graphene for LIBs. Herein, a laser‐induced strategy employing bituminous coal as a precursor for the preparation of porous graphene‐based materials (LIG‐B) is reported. LIG‐B exhibits a porous foam‐like structure and an enlarged interlayer spacing, which is larger than that of graphene with typical AB stacking. As the anode for LIBs, the LIG‐B shows a high specific capacity of 400 mAh g^−1^ at the current density of 100 mA g^−1^, and up to 95.0% of the initial reversible capacity retention after 900 cycles at 100 mA g^−1^. This result is higher than that of graphene‐based materials such as N‐doped rGO (200 mAh g^−1^), N‐doped Graphene film (150 mAh g^−1^), and rGO film (80 mAh g^−1^). Most importantly, a high capacity of 220 mAh g^−1^ can be maintained at 2000 mA g^−1^, indicating its superior rate capability. This work provides a low‐cost method to synthesize porous graphene‐based materials with fast Li^+^/electronic conductivity for high‐performance LIBs.

## Graphical abstract

A one‐step laser‐induced strategy is reported to prepare porous graphene by utilizing bituminous coal as a precursor (LIG‐B). Laser irradiation transforms coal into graphene, with small molecules from the polymer separating, recombining in gas and forming 3D porous graphene upon quick gas release. As a lithium‐ion battery (LIB) anode, LIG‐B ensures excellent rate and cycle performance.

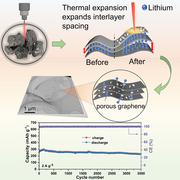

## 1 Introduction

Lithium‐ion batteries (LIBs) have been increasingly becoming the power source for electronic devices and are making significant inroads into sectors like low‐emission vehicles and energy storage systems due to their high energy density, long‐life cycles, flexible and lightweight design, and excellent environmental compatibility.^[^ ^1^, ^2^, ^3^, ^4^, ^5^, ^6^ ^]^ The performance of LIBs is predominantly governed by the anode materials, and the commercial available graphite anode suffers from the low theoretical capacity of 372 mAh g^−1^ and poor rate performance, which cannot meet the urgent demands for high energy storage systems utilized in practical applications.^[^ ^7^ ^]^ Therefore, it is highly desired to develop a novel anode material that are not only cost‐effective but also boast high specific capacities and enhanced safety features, aligning with the evolving demands of advanced energy storage systems.

Graphene was recognized as the most promising potential anode material for LIBs due to its high specific surface area, excellent electrical conductivity and superb electrochemical stability.^[^ ^8^, ^9^ ^]^ However, the current process for producing graphene is complicated and the precursor is difficult to obtain and expensive. Furthermore, the current capabilities of the graphene anode material of lithium‐ion battery in regard to fast charging do not align with the demands of the market. Coal, an amorphous polymer with a 3D cross‐linked network structure, is composed of aromatic rings, a small amount of hydrogenated aromatic rings, and other heterocycles connected by short aliphatic chains and ether bonds.^[^ ^10^, ^11^, ^12^ ^]^ As one of the most important atoms in coal, the carbon atom can form sp, sp^2^, and sp^3^ chemical bonds through different hybridizations, and then transform into different types of carbon materials, such as activated carbon, soft carbon, hard carbon, graphite, graphene and a variety of carbon nanomaterials.^[^ ^13^ ^]^ The microstructure of porous graphene is not only dependent strongly on the raw materials but also on the preparation routes, and the coal‐based graphene was mainly prepared by arc discharge method, chemical vapor deposition (CVD) and redox methods.^[^ ^14^, ^15^, ^16^ ^]^ Unfortunately, the complex preparation process and easy aggregation for 2D graphene caused by the strong van der Waals interactions among the nanosheets affect the high reversible capacity and large‐scale application for LIBs.^[^ ^17^, ^18^ ^]^ In recent years, laser processing, a versatile tool, has made notable advancements in fields like fine material processing and additive manufacturing due to its simple operation, high efficiency, wide applicability, and environmental benefits.^[^ ^19^ ^]^ Laser‐induced heating technology uses laser irradiation for rapid, spatially‐resolved heating of micro‐scale surfaces, enabling real‐time parameter modulation without chemicals for environmental sustainability. In addition, laser can induce photochemical and photothermal reactions, which may endow materials with novel structure and properties.^[^ ^20^, ^21^, ^22^ ^]^ Previously, Lin^[^ ^23^ ^]^ et al. and Ye^[^ ^24^ ^]^ et al. utilized a laser as a heat source and successfully synthesized porous graphene materials from polyimide and wood. However, polyimide is expensive and low laser penetration limits graphene film thickness (<1 mm). Although lignocellulose abounds in natural wood made up of cellulose, hemicellulose, and lignin,^[^ ^25^ ^]^ their distinct thermal decomposition behaviors cause uneven local reactions during laser carbonization, increasing graphene structural defects.

Herein, we report a one‐step laser‐induced strategy to prepare porous graphene‐based materials as anode material for LIBs by utilizing bituminous coal as a precursor. The coal was transformed into amorphous carbon upon the laser irradiation, and the more intense the ablation, the more the amorphous carbon was transformed into graphene. Meanwhile, the separating discrete small molecules or atoms from a polymer and then recombining them in a gaseous state occurs, and the 3D porous graphene was finally prepared due to the quick release of the gases. As the anode for LIBs, the as‐prepared porous graphene shows a fast Li^+^ transfer ability and good structural stability, thus leading to the good rate and cycle performance of the battery. This work provides a new quick way to regulate the micro‐structure of the porous graphene from low‐cost coal and achieve the stable and superior rate capability for LIBs.

## 2 Results and Discussion

**Figure 1:**
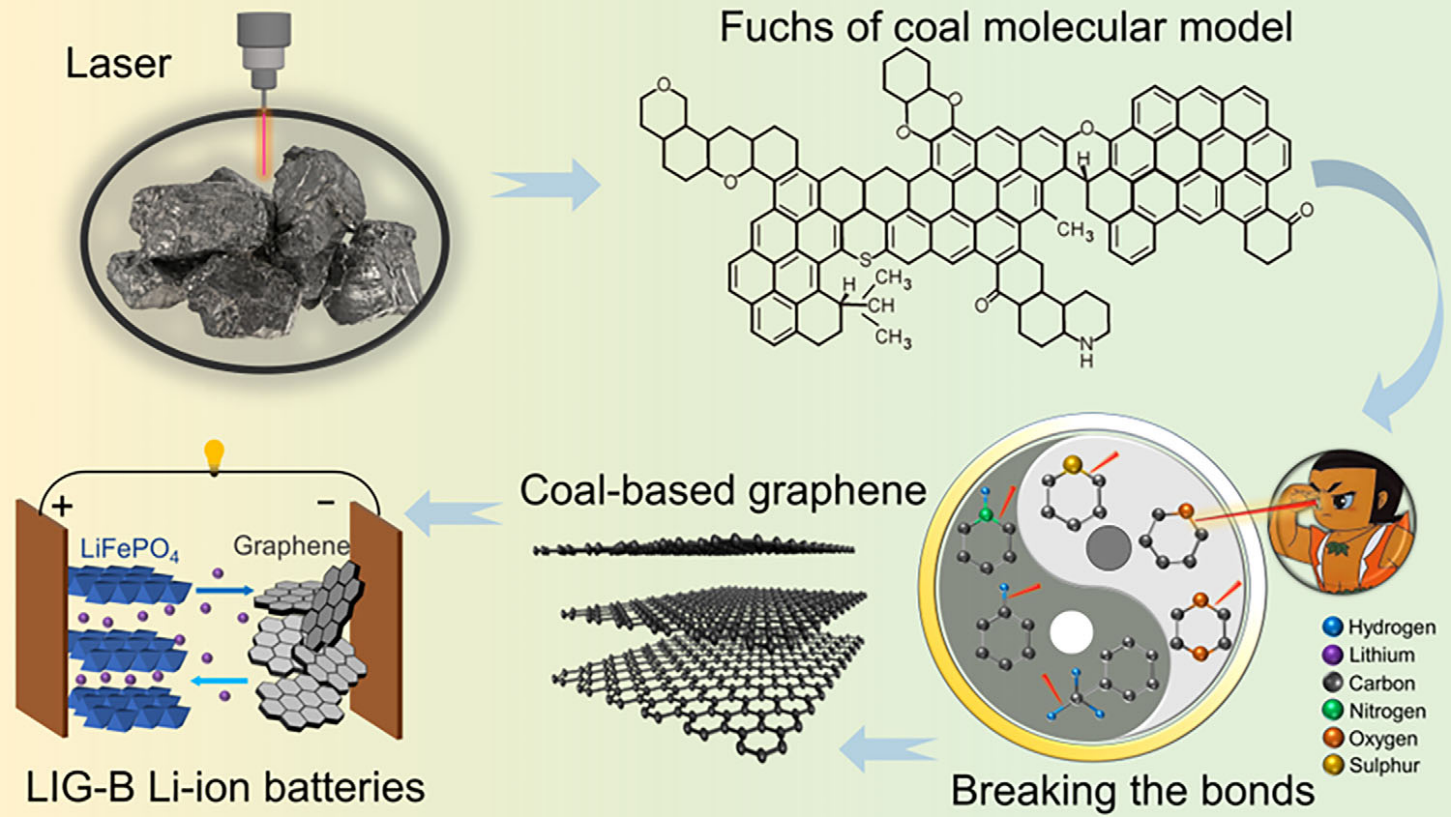
Schematic diagram of the preparation process of LIG‐B coal‐based graphene.

Graphene‐based materials were produced by laser‐induced process using bituminous coal as a precursor (denoted as LIG‐B). Table S1 (Supporting Information) shows the results of proximate and ultimate analyses of the LIG‐B coal sample. **Figure** **1** shows the preparation process of porous graphene‐based materials from bituminous coal (the Fuchs molecular model of bituminous coal^[^ ^26^ ^]^) using a laser irradiation method. The principle of laser‐induced generation of graphene is the photothermal effect, which refers to the process of converting light energy into heat. The energy generated from the laser irradiation causes the vibration of the lattice of precursor in the irradiated region, and the high temperature leads to the breaking of C─O, C═O, and N─C bonds, resulting in the rearrangement of carbon atoms to form the graphene structure.^[^ ^23^, ^24^, ^27^ ^]^ The process of laser irradiation first transforms the carbon precursor into amorphous carbon, and the additional ablation intensity further transforms the amorphous carbon state into graphene,^[^ ^28^ ^]^ in accompany with separating discrete small molecules or atoms from a polymer and then recombining them in a gaseous state. The quick release of the gases causes the formation of a porous structure in the graphene, which finally leads to a one‐step in situ synthesis of 3D porous graphene‐based materials.

**Figure 2:**
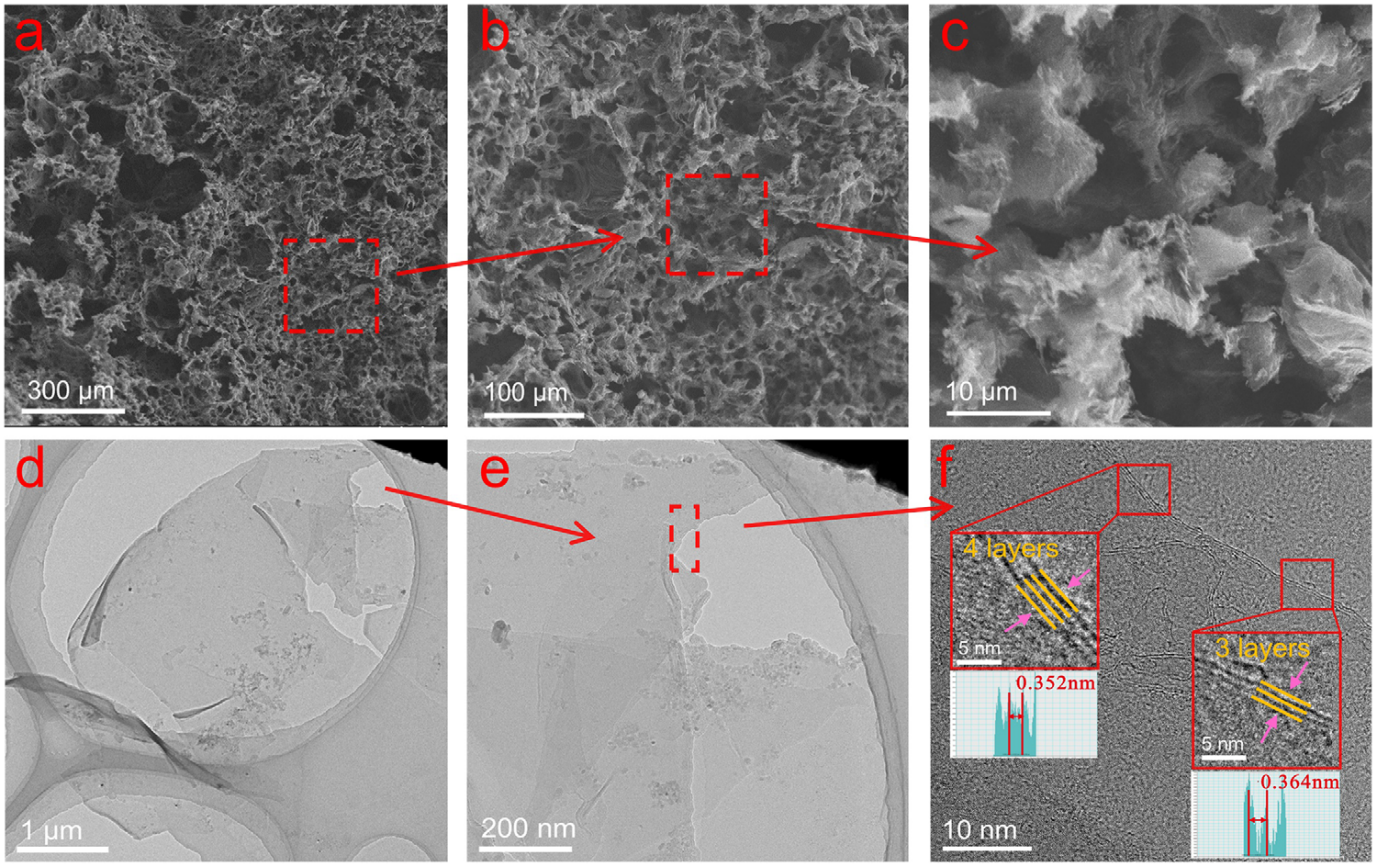
a–c) SEM images of LIG‐B at different scales. d–f) TEM images of LIG‐B at different scales.

As shown in **Figure** **2**a–c, scanning electron microscope (SEM) images of various resolutions reveal the porous foam‐like structure of LIG‐B. The center and edges of LIG‐B have numerous onion‐like pores with a lava morphology and an ellipsoid‐like shape. It can be attributed to the fact that the temperature of the laser‐irradiated area of the coal rises rapidly, thereby achieving maximum cracking of C─O, C═O, N─C, and C─H bonds and producing a large number of gas byproducts (CH_4_, NO_2_, N_2_, etc.).^[^ ^29^ ^]^ The rapid escape of gases due to thermal expansion leads to the formation of porous graphene structures. Notably, curled and folded graphene‐like structures are observed at the 10 µm scale (Figure 2c). As illustrated in Figure 2d,e, the transmission electron microscopy (TEM) images demonstrate the curling and interlayer stacking of LIG‐B. The interlayer spacing is found to be 0.364 nm for 3 layers graphene, and is 0.352 nm for 4 layers graphene (Figure 2f). These both two values are larger than the typical interlayer spacing of Bernal graphite (AB‐stacked), which is determined to be 0.337 nm.^[^ ^30^ ^]^ A large *d*_002_ in the graphene layer was reported to be capable of improving both the charge/discharge capacity and the coulombic efficiency of the battery due to the increased accommodation area of ions,^[^ ^31^ ^]^ thus, the larger interlayer spacing of graphene by laser‐ irradiated process for coal positively reflects the good lithium storage capacity.

**Figure 3:**
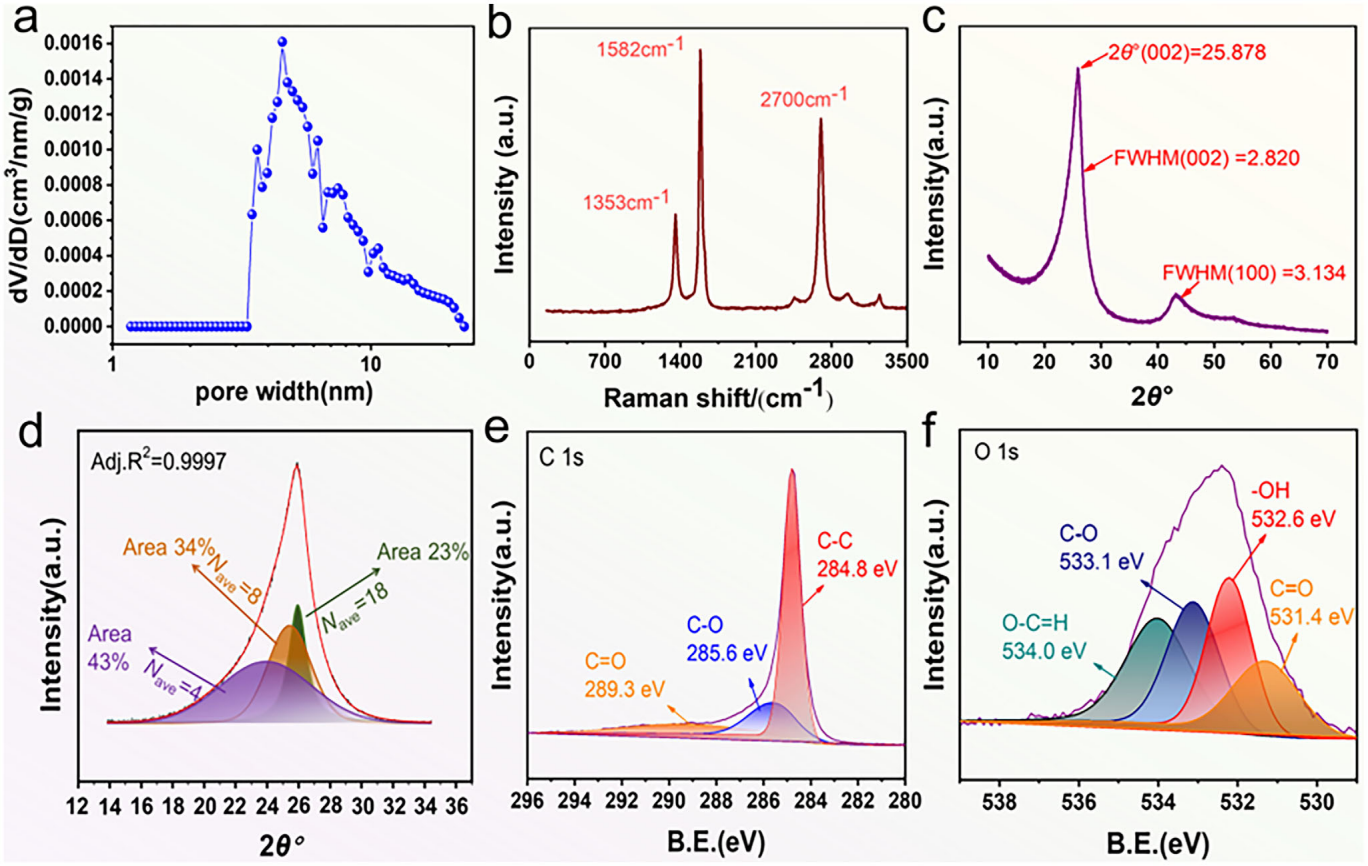
a) PSD of LIG‐B. b) Raman spectra of LIG‐B. c) XRD patterns of LIG‐B. d) Peak fitting results from LIG‐B. e,f) high‐resolution XPS spectra of C1s and O1s.

The pore size distribution (PSD) of LIG‐B were investigated by Brunauer‐Emmett‐Teller (BET) test, and the results were subsequently analyzed using the Barrett‐Joyner‐Halenda (BJH) method.^[^ ^32^ ^]^ As shown in **Figure** **3**a, the pore size ranges from 1.17 to 22.95 nm with the average pore size of 16.77 nm and the peak pore size of 4.54 nm, indicating the dominance of mesoporous pores in LIG‐B. The nitrogen adsorption and desorption result (Figure S1, Supporting Information) shows a type III adsorption isothermal curve, and a small H_3_‐type loop of the P/P_0_ ranging from 0.80 to 0.90 Pa can be observed, which is frequently observed in laminar‐structured aggregates that yield slit mesoporous or microporous materials, demonstrating the disordered mesoporous pore structure of the LIG‐B.^[^ ^33^ ^]^ In addition, at a pressure of 0.90 Pa, the total pore volume increases significantly, indicating the presence of macropores, which is consistent with SEM observations. The characteristic peaks of Raman spectrum of LIG‐B at ≈1353, ≈1582, and ≈2700 cm^−1^ can be designated to D, G, and 2D peaks, respectively (Figure 3b). the D peak at 1353 cm^−1^ induced by defects or bent sp^2^ carbon bonds, the first‐order allowed G peak at 1580 cm^−1^ and the 2D peak at 2700 cm^−1^ originating from second order zone‐boundary phonons.^[^ ^34^ ^]^ The intensity ratio I_D_/I_G_ of the D and G peaks in Raman spectroscopy can be used to characterize defect density of graphene.^[^ ^35^ ^]^ The I_D_/I_G_ ratio for LIG‐B is 0.47, indicating lower defect concentration. Furthermore, LIG‐B has a distinct 2D peak with I_2D_/I_G_ of 0.73, which suggests that the LIG‐B exhibits a few‐layer graphene structure,^[^ ^36^ ^]^ which is consistent with the results observed by HRTEM.

The X‐ray diffraction (XRD) diffraction peak at 25.878° in Figure 3c can be assigned to the (002) peak of the graphene, and the Full‐width‐at‐half‐maximum (FWHM) is 2.820. Based on the FWHM of (100) peak (3.134), Bragg's equation and Scheele's formula,^[^ ^37^, ^38^, ^39^ ^]^ the crystal size L_c_ along the c‐axis and the domain size L_a_ along the a‐axis of LIG‐B are calculated to be 2.859 and 5.573 nm, respectively. The L_a_/L_c_ value is also calculated to be 1.950, indicating that the transverse dimension of the crystal structure of LIG‐B is larger than the stacking thickness. The asymmetric (002) peak indicating the presence of different types of crystal structures within the LIG‐B.^[^ ^40^ ^]^ In order to further analyze the crystal structure in LIG‐B, the Voigt‐Lorentz‐Doppler (VLD) function was used to fit the (002) peak. The (002) peak can be divided into three peaks with d‐spacing of 0.340, 0.350, and 0.372 nm, corresponding to 18, 8, and 4 layers, respectively, and the peak area ratio indicates that ≈ 77% of the layers contain fewer than 10 layers, which suggests a substantial presence of few‐layer graphene and its predominant proportion (Figure 3d). This finding is consistent with the results observed by HRTEM and Raman.

X‐ray photoelectron spectroscopy (XPS) was employed to further elucidate the chemical nature of the LIG‐B, and C1s at 284.8 eV was used as a reference to calibrate the XPS binding energy (Figure 3e,f). As can be seen in Figure S2 (Supporting Information), the laser‐induced LIG‐B samples have a predominantly carbon (94.97%) and oxygen (5.03%) elemental composition, with the absence of other heteroatoms doping. Other elements in the coal, such as nitrogen and sulfur, form gases and escape during the laser‐induced process, suggesting that LIG‐B has a relatively low defect density, which is consistent with the observations from XRD and Raman spectroscopy. As illustrated in Figure 3e, the C1s high‐resolution spectrum evinces the presence of assorted carbon‐containing functional groups, including C─C (284.8 eV), C─O (285.6 eV), and C═O (289.3 eV). As shown in Table S2 (Supporting Information), the dominant C─C group suggesting that the LIG‐B primarily consisted of sp^2^ carbons. Meanwhile, the linear asymmetry of the C─C peak^[^ ^41^ ^]^ demonstrating that laser‐induced can successfully advance the graphitization of LIG‐B.^[^ ^42^ ^]^ As illustrated in Figure 3f, the O 1s high‐resolution spectra demonstrates that the oxygen‐containing functional groups of LIG‐B include C─O (533.1 eV), ‐OH (532.6 eV), O─C═H (534.0 eV), and C═O (531.4 eV). The C─O and ─OH groups in LIG‐B are present in the form of ether, alcohol, and O─C═H and C═O may originate from some amount of carbonyl, aldehyde, and ketone functionality (Table S2, Supporting Information). The presence of these oxygen‐containing functional groups has been demonstrated to be capable of improving the affinity between surfaces and ions and thus increasing the storage capacity of lithium ions batteries.^[^ ^43^ ^]^

**Figure 4:**
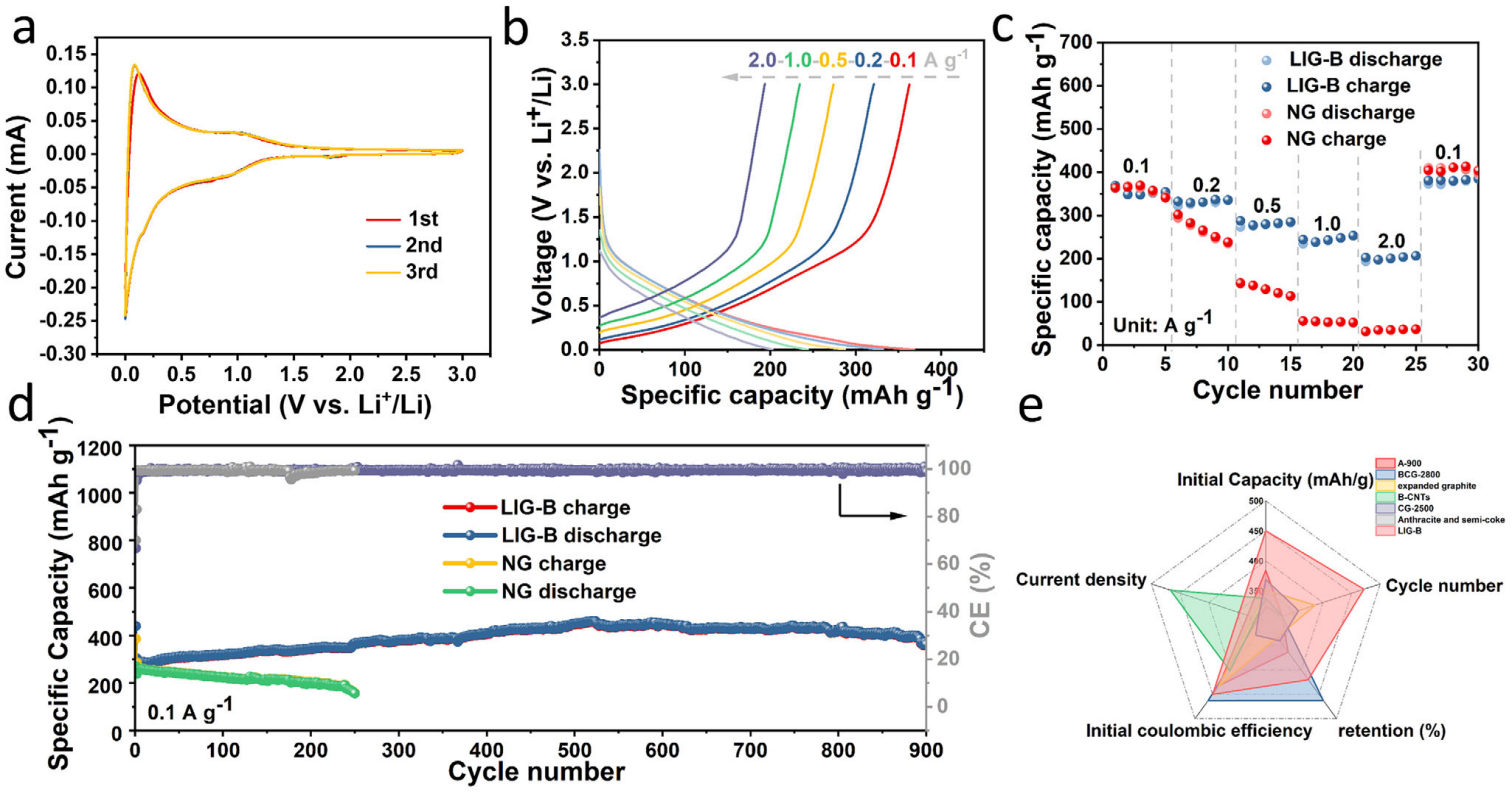
a) CV curves of LIG‐B at the scan rate of 0.1 mV s^− 1^. b) Charge‐discharge curves of LIG‐B at different current densities. c) Rate capability of LIG‐B and NG at current densities from 100 to 2000 mA g^−1^ for LIB. d) Cycling performances of LIG‐B and NG at current density of 100 mA g^−1^; e) Performance comparison of the LIG‐B anode with the previously reported coal carbon anode materials.

The electrochemical performance of the LIG‐B and NG (Natural graphite) were investigated to evaluate the application potential for LIBs. CR2032 coin cells were assembled with the LIG‐B and the NG as the anode materials and the lithium foil as reference electrode. **Figures** **4**a and S3 (Supporting Information) displays the CV curves of the first three cycles of the batteries with LIG‐B and NG anode. The reduction peak at 0–0.2 V observed in the cell with LIG‐B can be attributed to the intercalation of lithium ions into LIG‐B carbon. A corresponding oxidation peak appeared at 0.1–0.2 V can be attributed to the delithiation process.^[^ ^44^ ^]^ The CV curves of the three cycles almost overlap, suggesting the good stability and reversibility of LIG‐B anode. It should be noted that the small oxidation peak at about 0.70 V in the first cycle is resulted from the electrolyte decomposition and the formation of solid electrolyte interface (SEI) film.^[^ ^45^ ^]^ According to Faraday's first law,^[^ ^46^ ^]^ a larger CV curve of LIG‐B indicates a larger amount of oxidized or reduced species and a larger amount of charge generated by the reaction, thus implying a large electrochemical surface area of the electrode. As for the cell with NG, the CV curves display the sharp peaks between 0 and 0.5 V, which is ascribed to the Li^+^ insertion/desertion in the lamellar graphite (Figure S3, Supporting Information). In addition, the CV curves of cell with LIG‐B anode shows no obvious sharp peaks, which are consistent with the charge‐discharge curves (Figure 4b).^[^ ^47^ ^]^ The cell with LIG‐B anode show greatly enhanced rate performances than that of the cell with NG anode (Figure 4c). LIG‐B anode delivers the most impressive rate capability with a high discharge capacity of 220 mAh g^−1^ at 2000 mA g^−1^. When the current density goes back to 100 mA g^−1^, the discharge capacity of LIG‐B can be restored to 400 mAh g^−1^, indicating the good electrochemical reversibility of LIG‐B. Whereas, for the cell with NG anode, when the current density is higher than 1000 mA g^−1^, the specific discharge capacity is almost reduced to 0 mAh g^−1^. It is due to the large layer spacing and abundant mesoporous structure of the LIG‐B, which can facilitate the transportation rate of Li^+^ and enable the fast electrochemical reaction.

As expected, the cell with LIG‐B anodes also deliver superior cycle stability (Figure 4d). The discharge‐specific capacity of LIG‐B is 400 mAh g^−1^ at the current density of 100 mA g^−1^, and it retains 95% of its capacity after 900 cycles, suggesting a good cycle stability. By contrast, the cell with NG anodes shows a significant capacity fading after 200 cycles with the capacity retention of only 66%. Considering the practical application, the long‐cycle performance at 2000 mA g^−1^ were further investigated. As shown in Figure S5 (Supporting Information), the capacity retention of the cell with LIG‐B anode still maintains at 200 mAh g^−1^ after 3500 cycles with the capacity retention of 90%, indicating the outstanding long‐cycle performance of LIG‐B. It should also be mentioned that the electrochemical performance of LIG‐B anode is much better than many other carbon materials reported in literatures (Figure 4e; Table S3, Supporting Information). Specifically, the good cycle stability of LIG‐B anode can be mainly ascribed to the porous structure, which can relieve the volume expansion during the repeated charge‐discharge process.

**Figure 5:**
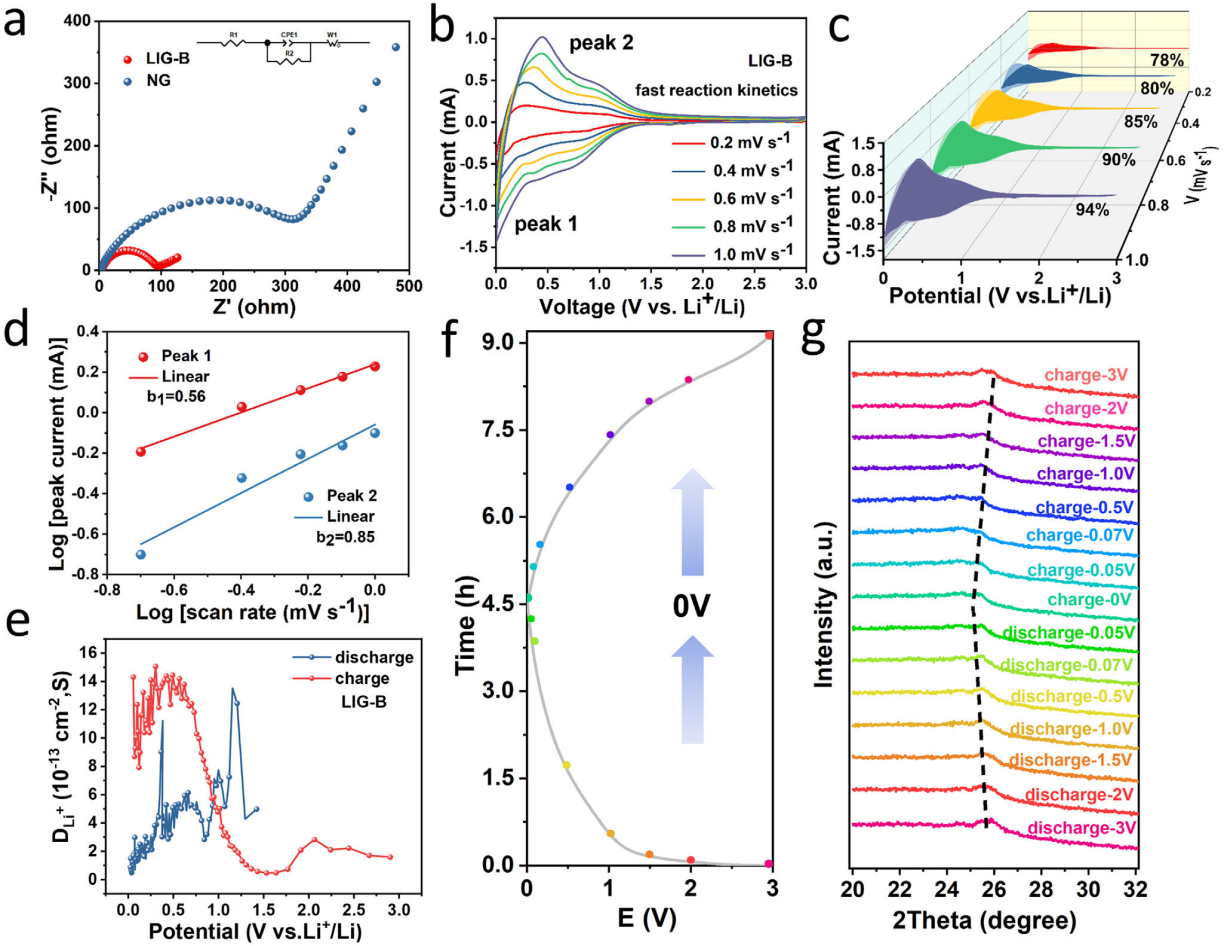
a) EIS of the batteries with the two anodes. b) CV curves of LIG‐B at different scan rates from 0.2 to 1 mV s^−1^. c) Capacitive charge‐storage contributions at different scan rates from 0.2 to 1 mV s^−1^. d) Relationship between the peak current and scan rate. e) The calculated D _Li+_ of LIG‐B varies with potential. f) Time‐voltage curve. g) In situ XRD patterns of LIG‐B during discharge–charge.

The kinetic parameters of the anodes were further analyzed by the Nyquist plot. As shown in **Figure** **5**a, the plots of the two electrodes all consist of a semicircle (high frequency area) and an oblique straight line (low frequency area). In the high‐frequency region of the impedance spectrum, the intersection of the semicircle with the transverse axis represents the ohmic resistance (R_s_), while the diameter of the semicircle is related to the charge transfer impedance (R_ct_) at the electrolyte‐electrode interface. The charge‐transfer resistance (R_ct_) of LIG‐B is 90 Ω, which is much lower than that of NG (318 Ω), indicating the fast charge transfer in the LIG‐B anode. In addition, the diffusion coefficient of Li^+^ can be calculated from the low frequency region of EIS spectra based on the following Equation (1):^[^ ^48^ ^]^

(1) $$D_{Li^{+}}=0.5{\left(RT/An^{2}F^{2}\sigma_{w}CL_{i}\right)}^{2}$$

where, R, T, A, n, F and C_Li_ are gas constant, Kelvin temperature, surface area of working electrode, number of electrons of each molecule involved in the reaction, Faraday constant and Li^+^ concentration, respectively. The Warburg factor (σ_w_) is related to Z′ according to Equation (2):^[^ ^49^ ^]^

(2) $$Z^{\prime }=R_{e}+R_{ct}+\sigma_{w}w^{-\frac{1}{2}}$$

The relationship between Z^’^ and the square root of the frequency (*w* ^−1/2^) was shown in Figure S6 (Supporting Information). From the slope of the fitting line in Figure S6 (Supporting Information) and Equation (2), the Li^+^ diffusion coefficient of LIG‐B was calculated, that was 4.15 × 10^−12^ cm^2^ s^−1^, which was much higher than that of the reference one (7.58 × 10^−14^ cm^2^ s^−1^). The results were consistent with the CV analysis. The Li^+^ storage mechanism in LIG‐B anode was further analyzed by CV curve analysis at different scan rates (Figure 5b; Figure S11, Supporting Information). The Li^+^ storage mechanism mainly includes the capacitive effects and diffusion‐controlled intercalation‐based behavior which can be concluded by the following Equations (3) and (4):^[^ ^50^ ^]^

(3) $$i\left(v\right)=av^{b}$$

(4) $$\log\left(i\right)=\;\log\left(a\right)+b\log\left(v\right)$$

where *i* represents the peak current, *ν* is the sweep rate, *a* is the constant and *b* is the slope of the line as a function of *log (i)* and *log (v)*. And if the *b* value nears to 0.5, intercalation‐based behavior is the main storage mechanism of Li^+^. By contrast, if the *b* value nears to 1, capacitive effects will be the main storage mechanism of Li^+^ in the electrode. As shown in Figure 5d, the slopes (b_1_ and b_2_) obtained by fitting the peak 1 and peak 2 (Figure 5b) are about 0.56 and 0.85, indicating that the Li^+^ storage in LIG‐B electrode is controlled by diffusion‐controlled and capacitive effects, respectively. The capacitive‐controlled contribution in LIG‐B and NG were further investigated by analyzing the CV curves at various sweep rates from 0.2 to 1.0 mV s^−1^ (Figure 5c; Figure S12, Supporting Information). After calculation, the capacitive contribution of LIG‐B is 78% at a low scan rate of 0.2 mV s^−1^. And the proportion of capacitive contribution increases gradually with the increasing of sweep rate, and it reaches up to 94% at a high scan rate of 1.0 mV s^−1^ (Figure S7, Supporting Information), which is higher than that of NG at the same high sweep rate of 1.0 mV s⁻¹ (Figure S13, Supporting Information). The capacitive contribution in LIG‐B especially at high current density is beneficial for the improvement of rate performance of the battery.^[^ ^51^ ^]^ Subsequently, galvanostatic intermittent titration technique (GITT) tests were also conducted to analyze lithium‐ion storage behavior (Figures S8 and S9, Supporting Information). The corresponding diffusion coefficient of Li^+^ during one cycle was calculated by following Equation (5):^[^ ^52^ ^]^

(5) $$D=\frac{4}{\pi \tau }\;{\left(\frac{m_{B}V_{M}}{SM_{B}}\right)}^{2}{\left[\frac{\Delta E_{s}}{\Delta E_{t}}\right]}^{2}\;\left(t\ll \frac{L^{2}}{D}\right)$$

where τ is the pulse duration, m_B_ is the mass of active material, M_B_ is the molar mass of graphene, V_m_ is the molar volume of electrode material, S is the surface area of active material, ΔE_S_ is the potential change caused by the pulse, ΔE_τ_ is the potential change due to the pulse current. ΔE_S_ and ΔEτ can be obtained from GITT curves. Figure 5e presents the changing trends of D_Li+_ during one cycle, and the D_Li+_ of LIG‐B is larger than that of NG (Figure S10, Supporting Information). The difference of diffusion coefficients of Li^+^ between slope and plateau region also reveals two kinds of different storage mechanisms. The large D_Li+_ corresponds to fast adsorption process in slope region and the small D_Li+_ attributes to diffusion process in plateau region, respectively.

The in situ XRD was used to observe the structure evolution of the material during cycling, which can reflect Li^+^ insertion behaviors in host material. The (002) peak position changes not obviously in slope region, demonstrating that there is no Li^+^ inserted into interlayers. In contrast, with discharging in plateau region under 0.1 V, the (002) peak distinctly moves to lower angle, demonstrating a dilation of d_002_ distance (Figure 5f,g). The phenomenon confirms the adsorption‐intercalation mechanism in the discharging process and the gradual deintercalation during the whole charging process.^[^ ^53^ ^]^ More importantly, no obvious shift for the (002) peak can be observed during the charge/discharge process, which can be ascribed to the structural stability of LIG‐B, and the (002) plane has enough spacing (**0.372 nm**) to accommodate lithium ions, which enables the LIG‐B to deliver impressive cycle stability at large current density. The structural stability of LIG‐B was demonstrated by observing the morphological evolution of the electrode surface during cycling, and the NG and LIG‐B were characterized by SEM in the initial state and at 1000 mAh g^−1^ after 200 cycles (Figure S14, Supporting Information). The LIG‐B electrode maintains a smooth surface morphology and intact particles even after 200 cycles, whereas the NG electrode demonstrates large numbers of cracks over the whole electrode surfaces and severe pulverization (see inset of Figure S14e, Supporting Information). The emergence of the cracks is a symptom of the inefficient stress release under the strong volume change. The newly exposed surfaces of the cracks and divided smaller active particles in the electrode would consume more electrolytes for SEI formation.^[^ ^54^ ^]^ The gradual electrode structure degradation is the key reason for the low coulombic efficiency, significant capacity decay, and final battery failure.

**Figure 6:**
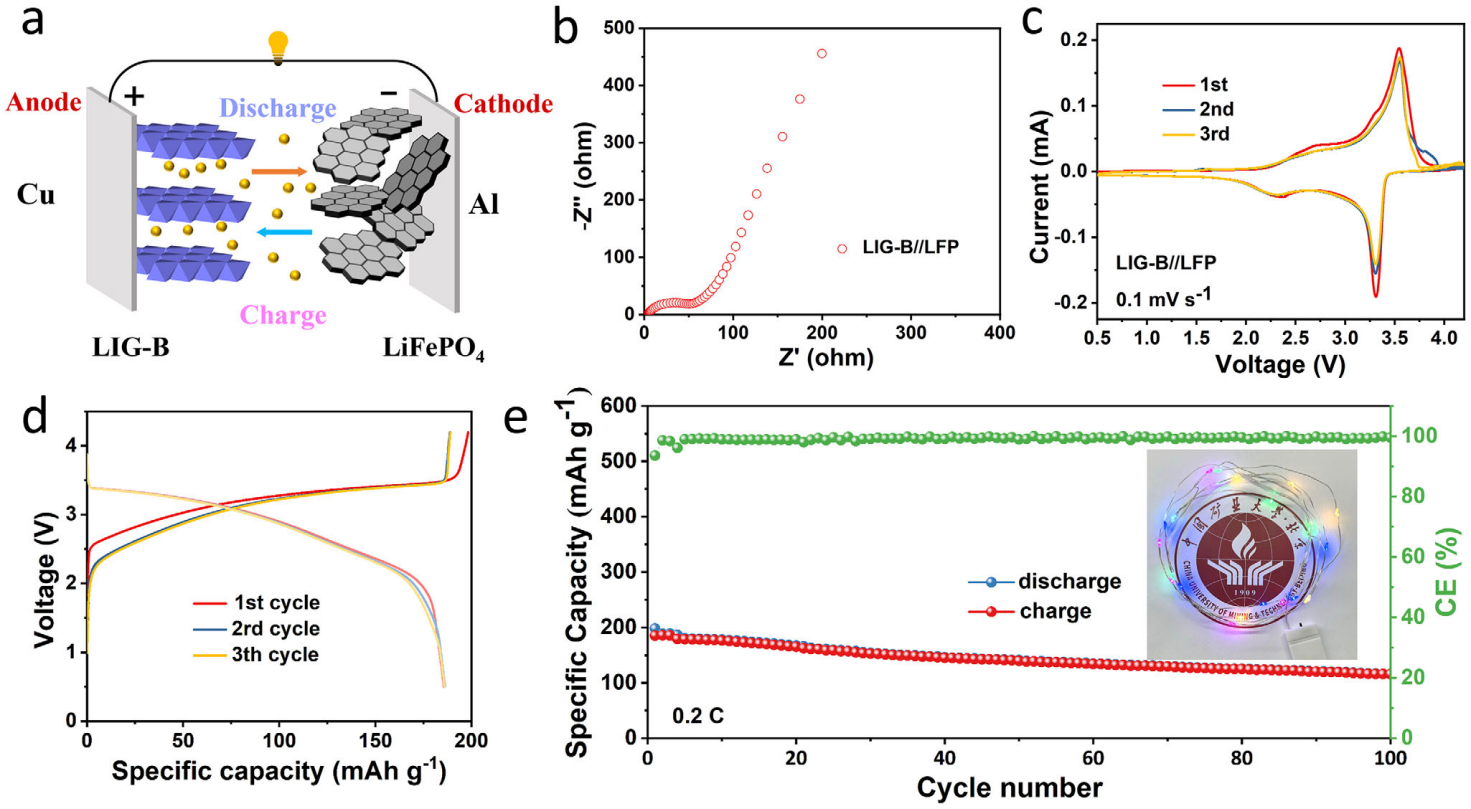
a) The schematic of coin full cell; b) EIS of the coin full cell; c) CV curves of coin full cell at the scan rate of 0.1 mV s^− 1^; d) The charge–discharge profiles of full cell; e) The cycle performance of full cell with an insert featuring a lit LED.

Furthermore, to evaluate the practical application ability of the anode materials, the full cells were assembled by using LiFePO_4_ as cathode materials and LIG‐B as anode material, as shown in **Figure** **6**a. The N/P ratio of the full cell is 1.1 and voltage range is set between 0.5 and 4.2 V. The charge‐transfer resistance (R_ct_) of LFP//LIG‐B is only 60 Ω (Figure 6b). The high coincidence of CV curves in Figure 6c indicates that the full cell also has high electrochemical reversibility. Figure 6d shows that the LiFePO_4_/LIG‐B full cell exhibits a high specific capacity of 190 mAh g^−1^ at 0.2 C (based on the mass of anode) with an average operation voltage of 3.2 V. As shown in Figure 6e, LiFePO_4_/LIG‐B delivers outstanding cycle performance with a high‐capacity retention ratio of 85% after 100 cycles. The practical application potential was further validated by powering a LED (inset of Figure 6e), highlighting its promising prospects. Meanwhile, LIG‐B//LFP pouch cells were assembled to illustrate the potential in large‐scale production. The open‐circuit voltage of the assembled pouch cells was tested using a multimeter and was found to be 2.67 V. (Figure S15, Supporting Information). Subsequently, the electrochemical performance tests were conducted under the conditions of a voltage range of 2.5–3.65 V at a current density of 0.2C. Figure S16 (Supporting Information) shows good cycling stability with 98% capacity retention after 50 cycles. Finally, the pouch cell can successfully charge the Digital watch (Figure S17, Supporting Information).

## 3 Conclusion

In conclusion, a porous graphene‐based materials anode was successfully prepared based on the laser‐induced strategy. The coal precursor transforms into amorphous carbon first, and then into graphene upon laser irradiation, and the 3D porous graphene‐based materials was obtained due to the quick release of the gases released from the precursor. The abundant mesopores and increased layer spacing of the porous graphene‐based materials can be beneficial for the diffusion and storage of lithium ions. As the anode for LIBs, the LIG‐B shows a high specific capacity of 400 mAh g^−1^ at the current density of 100 mA g^−1^. Most importantly, the high discharge capacity of 220 mAh g^−1^ can be obtained at the current density of 2000 mA g^−1^. The remarkable electrochemical performances indicated that coal‐based anode materials have good potential for application in advanced lithium‐ion batteries. This work will provide an effective and low‐cost strategy for designing a coal‐based anode for LIBs or other second batteries.

## 4 Experimental Section

### Preprocessing

Coal sample LIG‐B was crushed and ground to 75 µm for demineralization. The presence of a substantial quantity of inorganic mineral components in a highly complex state within the coal may have a detrimental impact on the final product. Therefore, coal samples were taken by HCl‐HF acid demineralization method, removing impurities and minerals from the sample.

### CO_2_ Laser‐Induced LIG‐B

The laser instrument, designated AWC708C (Universal Laser Systems), is a self‐assembled commercial 10.64 µm CO₂ infrared laser. Scanning rate of 15 cm s^−1^, the sample is scanned by controlling the irradiation time and regulating the power in accordance with the relevant specifications.

### Materials Characterization

The SEM images were examined by the Hitachi SU‐8020 high‐resolution field emission scanning electron microscope, with a magnification ranging from 40 to 80000 and an acceleration voltage of 0.5–30 kV. The measurement of N₂ adsorption/desorption isotherms were conducted using the Autosorb‐iQ instrument from Kantar, USA, and the fully automated physical adsorption analyzer ASAP2020 from Mack. The samples were subjected to a low‐temperature (77 K) analysis of their specific surface area and pore structure distribution, employing N₂ as the adsorbent. The Brunauer‐Emmett‐Teller (BET) method, with a relative pressure (P/P_0_) of 0.001‐1, is to be employed in order to calculate the pore volume of the material. The complete pore size distribution of the material was calculated using Density Functional Theory (DFT). TEM images were taken using the 200 kV JEOL‐JEM 2100F field emission gun TEM. Using Nicolet FT‐Raman 960 spectrometer with a 532 nm Ar laser excitation. The surface power is 5 mW, the number of accumulations is 10, and the integration time is 15 s. X‐ray diffraction is tested in powder form on the Bruker D8 ADVANCE diffractometer. The radiation source is a copper target (Cu‐Kα), wavelength λ = 1.54 Å, Tube pressure 40 kV and tube current 40 mA. The fixed step size is 0.02°, the scanning speed is 2°min^−1^, and the 2θ scanning range is 10°∼70°. XPS was tested on an American Thermo scalable 250 XI instrument. Relevant parameters: Monochromatic Al Kα (hv = 1486.6 eV), power 150 W, 650 µm beam spots, voltage 14.8 KV, current 1.6 A, Charge correction was performed using contaminated carbon C1s = 284.8 eV. The full spectral fluence was measured in steps of 1 eV, while the high‐resolution spectral fluence was measured in steps of 0.1 eV.

### XRD Calculations

Using the (002) peak integrals alongside Bragg's equation and Scherrer's formula, the (002) interplanar spacing (*d*_002_), average microcrystalline height (*L*_c_), microcrystalline diameter (*L*_a_), and mean number of aromatic layers (*N*_ave_) were calculated. The X‐ray wavelength λ = 1.54 Å. The full width at half maximum (FWHM) of the (002) and (100) crystalline diffraction peaks were denoted as *β*_002_ and *β*_100_, respectively, with corresponding diffraction angles *θ*_002_ and *θ*_100_ for these planes. The *k*1 and *k*2 represent the microcrystalline shape factors, *k*1 = 1.84 and *k*2 = 0.94.

(6) $$d_{002}=\frac{\lambda }{2\sin\theta_{002}}$$

(7) $$L_{a}=k_{1}\frac{\lambda }{\beta_{100}\cos\theta_{100}}$$

(8) $$L_{C}=k_{2}\frac{\lambda }{\beta_{100}\cos\theta_{100}}$$

(9) $$N_{ave}=L_{c}/d_{002}$$

### Electrochemical Measurement

Electrochemical performance were investigated in half‐cell system (CR2032) with metallic lithium as another electrode. The coin cells were assembled in a glove box filed with argon (Mikrouna). The anode was made from a slurry mixture containing an active material, Super P and polyvinyl difluoride (PVDF) binder (weight ratio is 8:1:1). Then the slurry mixture was pasted on a copper foil and dried at 60 °C for 12 h. Finally, the copper foil was cut to form the anode disc with diameter of 12 mm. According to the data provided by XPS in Figure S2 (Supporting Information), the carbon content of the sample is approximately 95%. Therefore, the mass of the active material in the negative electrode is calculated as follows: Mass of active material = (Electrode weight−Weight of copper foil) ×0.8×Carbon content = (9.7 mg−8.4 mg) ×0.8 × 95% = 1.064 mg. The active mass loading of the electrodes is 0.94 mg cm^−2^. The charge‐discharge performance of the batteries was tested with a Landt system (Wuhan). The cyclic voltammetry (CV) curves and Electrochemical Impedance Spectroscopic (EIS) spectra were investigated by Electrochemical Analyzer (CHI660E).

## Conflict of Interest

The authors declare no conflict of interest.

## Supporting information



Supporting Information

## Data Availability

The data that support the findings of this study are available from the corresponding author upon reasonable request.
