# Supplementary material for: Laser‐Induced Coal‐Based Porous Graphene as Anode Toward Advanced Lithium‐Ion Battery
Source: Adv Sci (Weinh). 2025 May 8;12(28):2504592. doi: 10.1002/advs.202504592 (PMC12302631; doi:10.1002/advs.202504592)
Supplement: Supplementary file 1 — Supporting Information [file ADVS-12-2504592-s001.docx]

Supporting Information

Laser-induced coal-based porous graphene as anode towards advanced lithium-ion battery

Xiao Ma ^a‡^, Shiyue Li ^b‡^, Wenhao Tang ^b^, Ruiping Liu ^b*^, Zilong Fu ^a^, Shaoqing Wang ^a*^


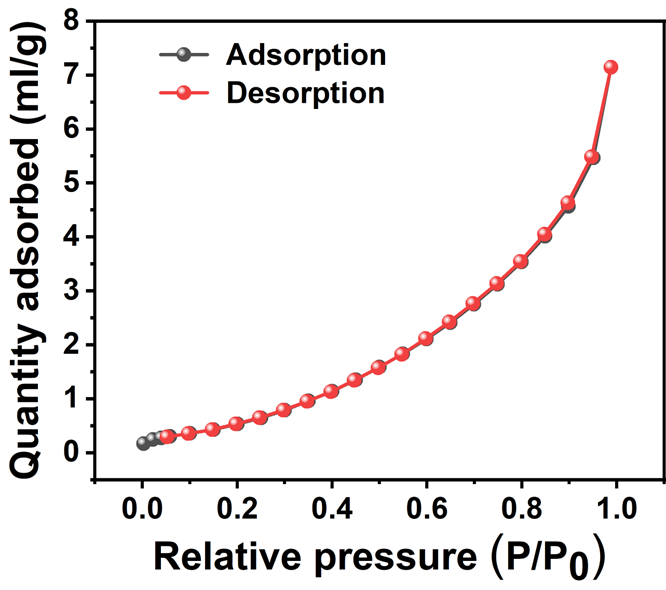


**Fig. S1**. LIG-B nitrogen adsorption and desorption curve.


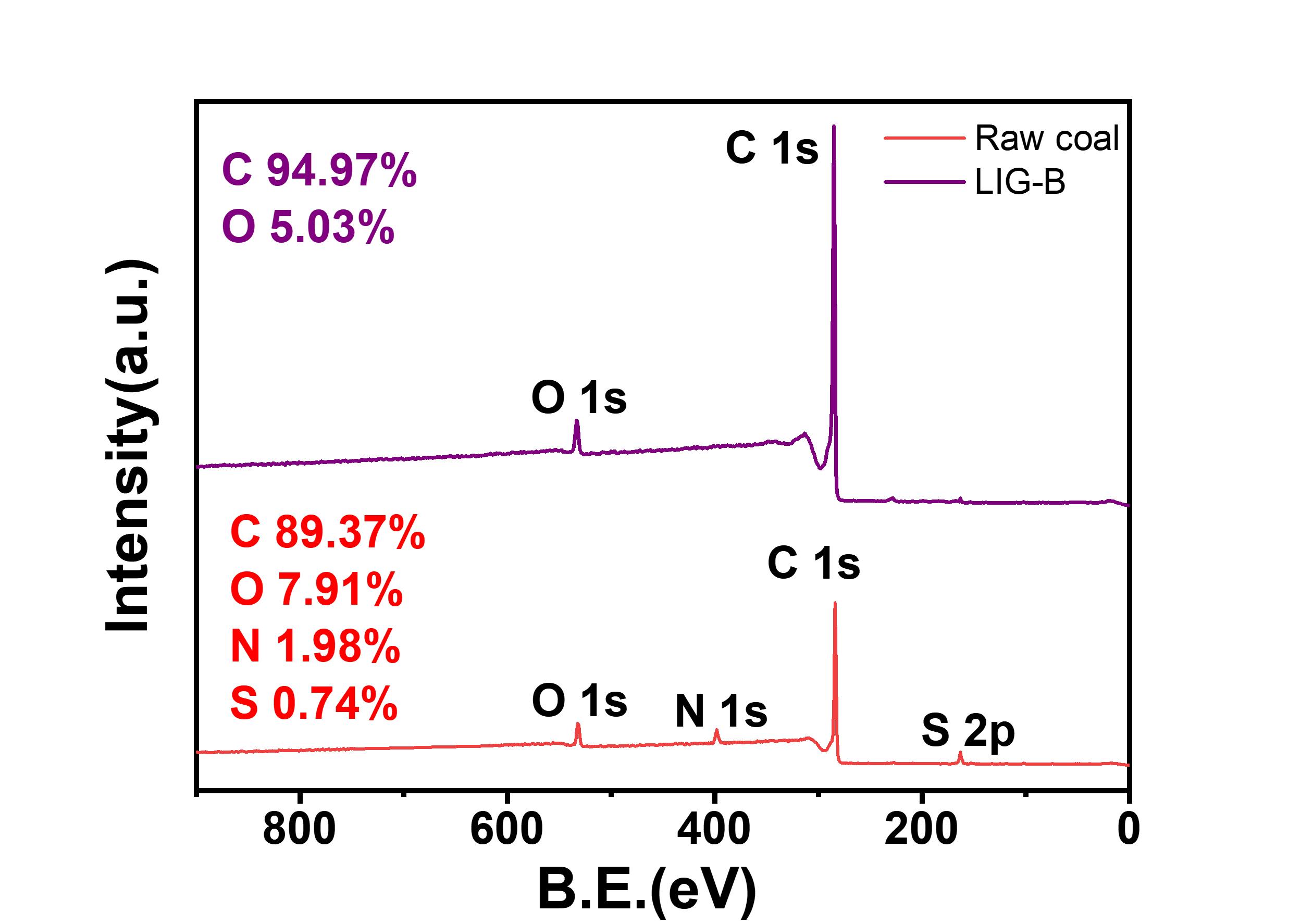


**Fig. S2.** The XPS of LIG-B and Raw coal of LIG-B.


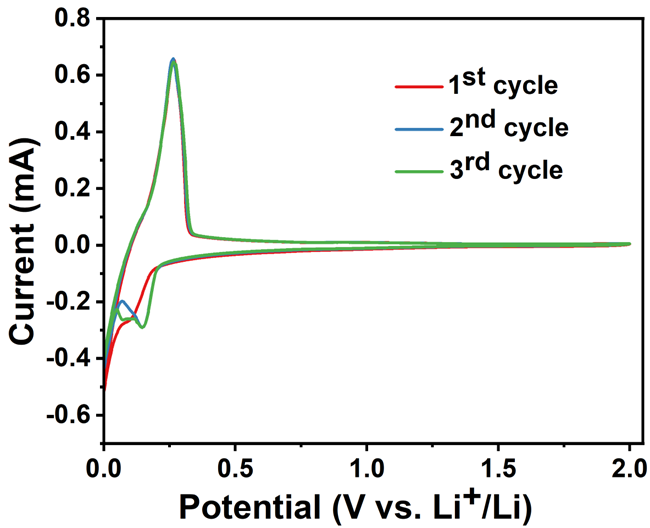


**Fig. S3.** CV curves of LIG-B at the scan rate of 0.1 mV s^− 1^.


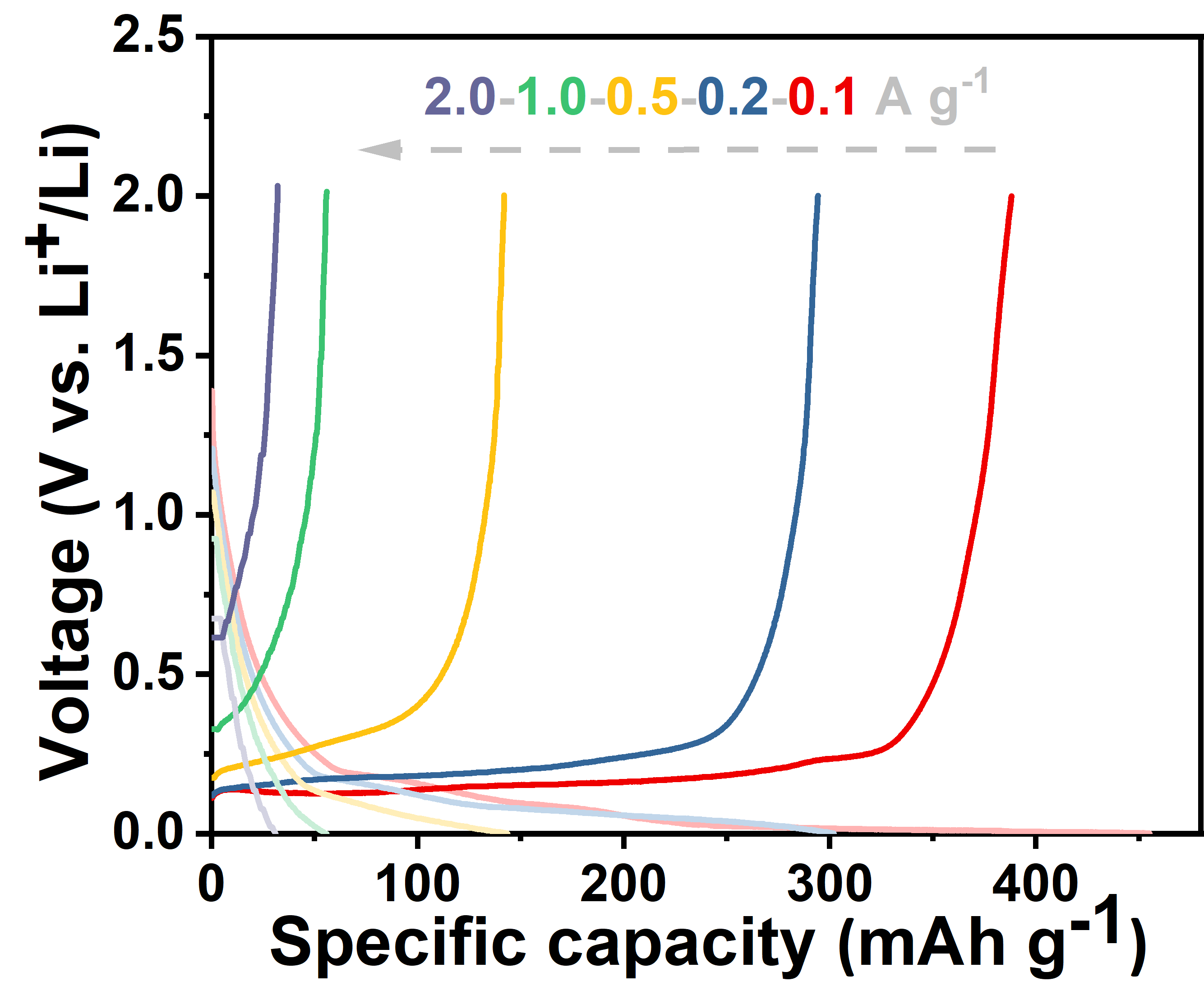


**Fig. S4.** Charge-discharge curves of NG at different current densities.

**Fig. S5.** Cycling performances of LIG-B at current density of 1000 mA g^-1^;


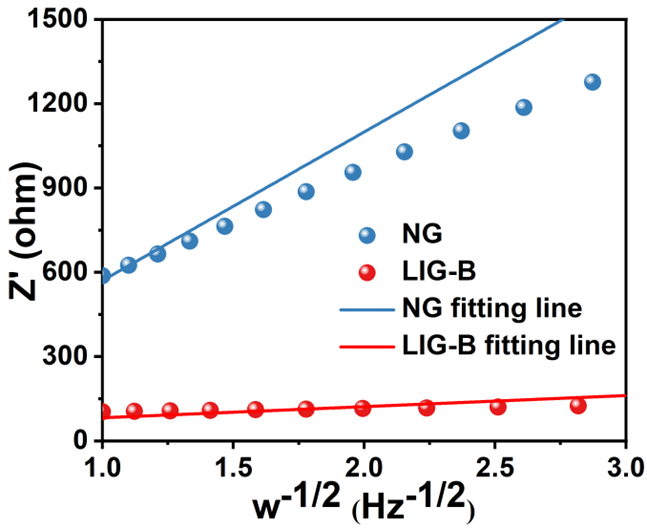


**Fig. S6.** Linear fitting of inclined line at low frequency;


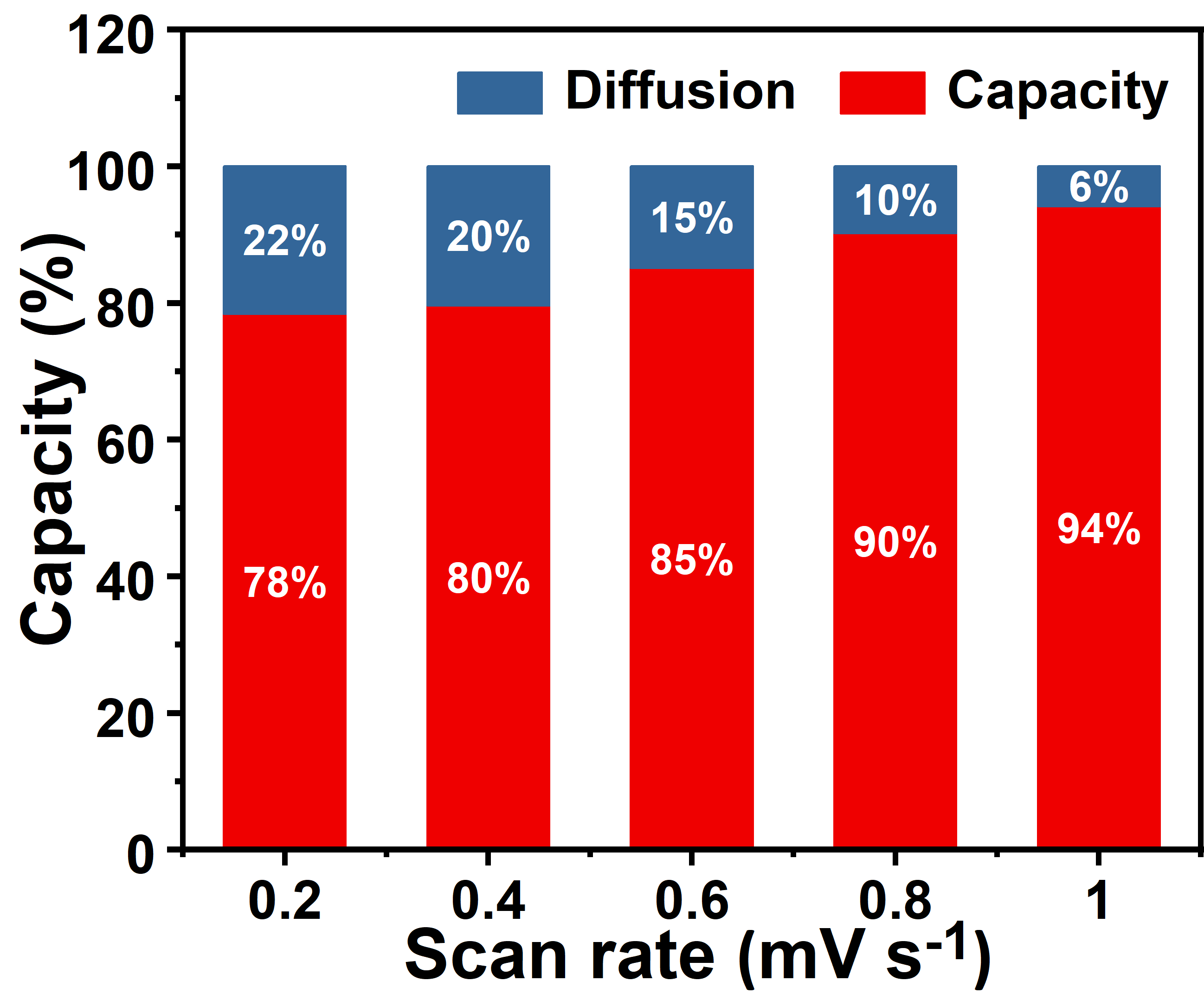


**Fig. S7.** Capacitive and diffusion-controlled capacity contributions for the LIG-B electrode at different scan rates;


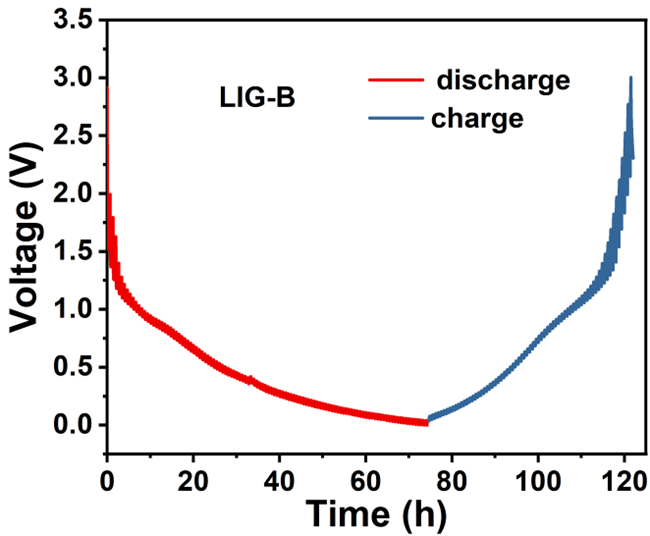


**Fig. S8.** GITT profiles of LIG-B;


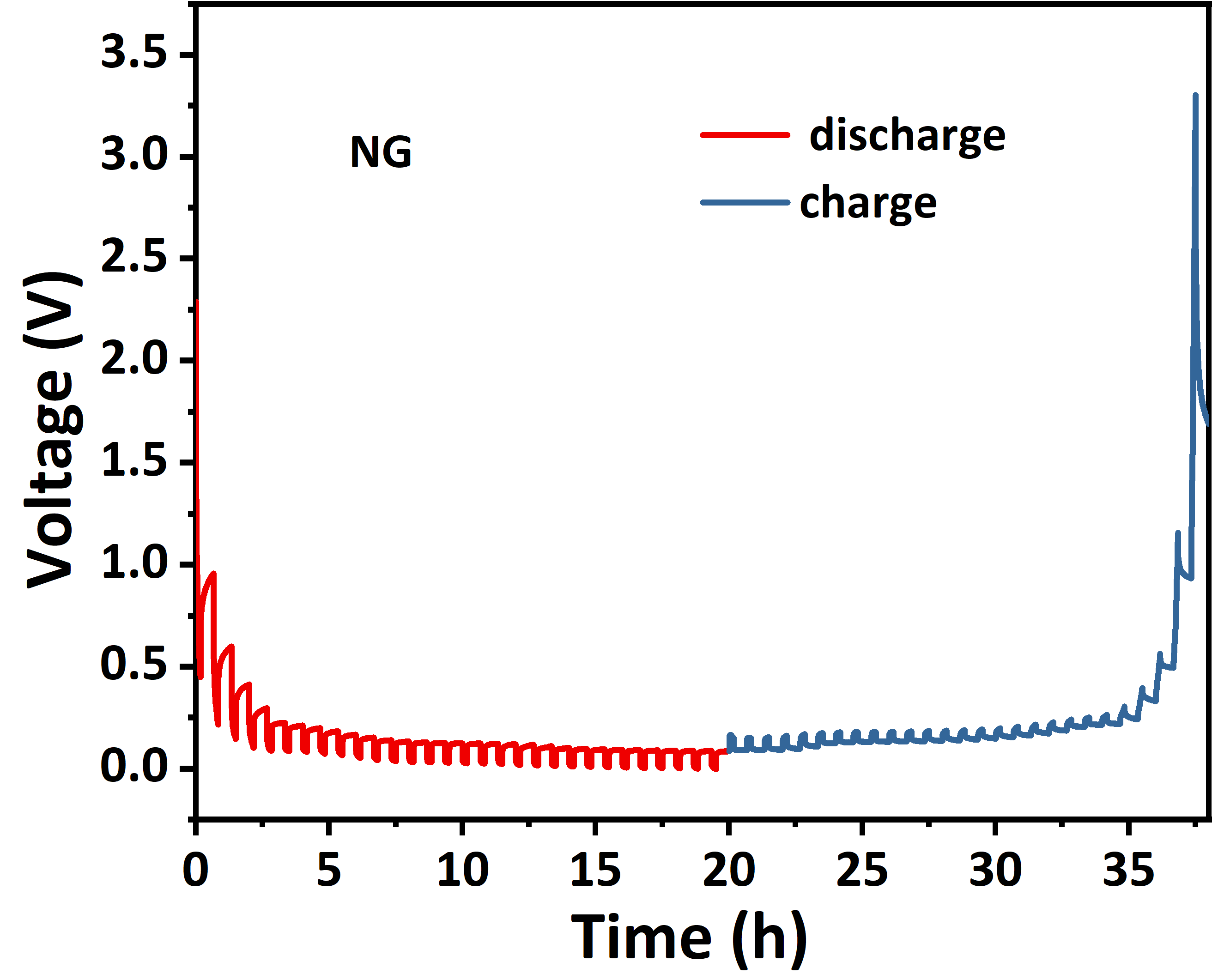


**Fig. S9.** GITT profiles of NG

**Fig. S10.** The calculated D _Li+_ of NG varies with potential.


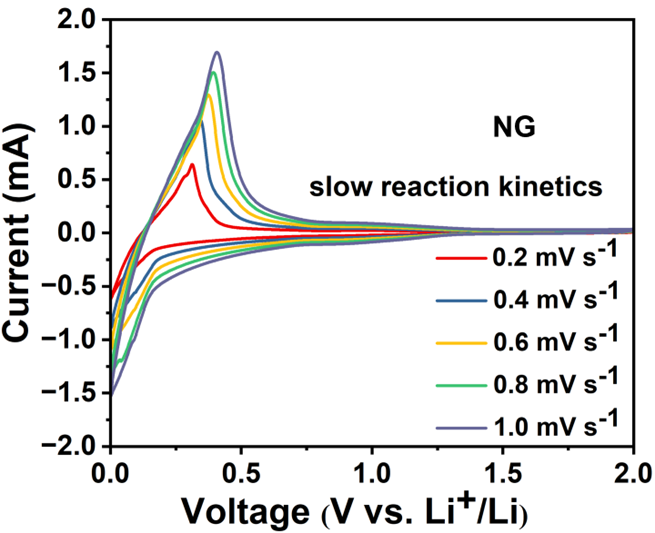


**Fig. S11**. CV curves of NG at different scan rates from 0.2 to 1 mV s^-1^.

**Fig. S12**. Capacitive charge-storage contributions for the NG electrode at different scan rates from 0.2 to 1 mV s^-1^.

**Fig. S13.** Capacitive and diffusion-controlled capacity contributions for the NG electrode at different scan rates.


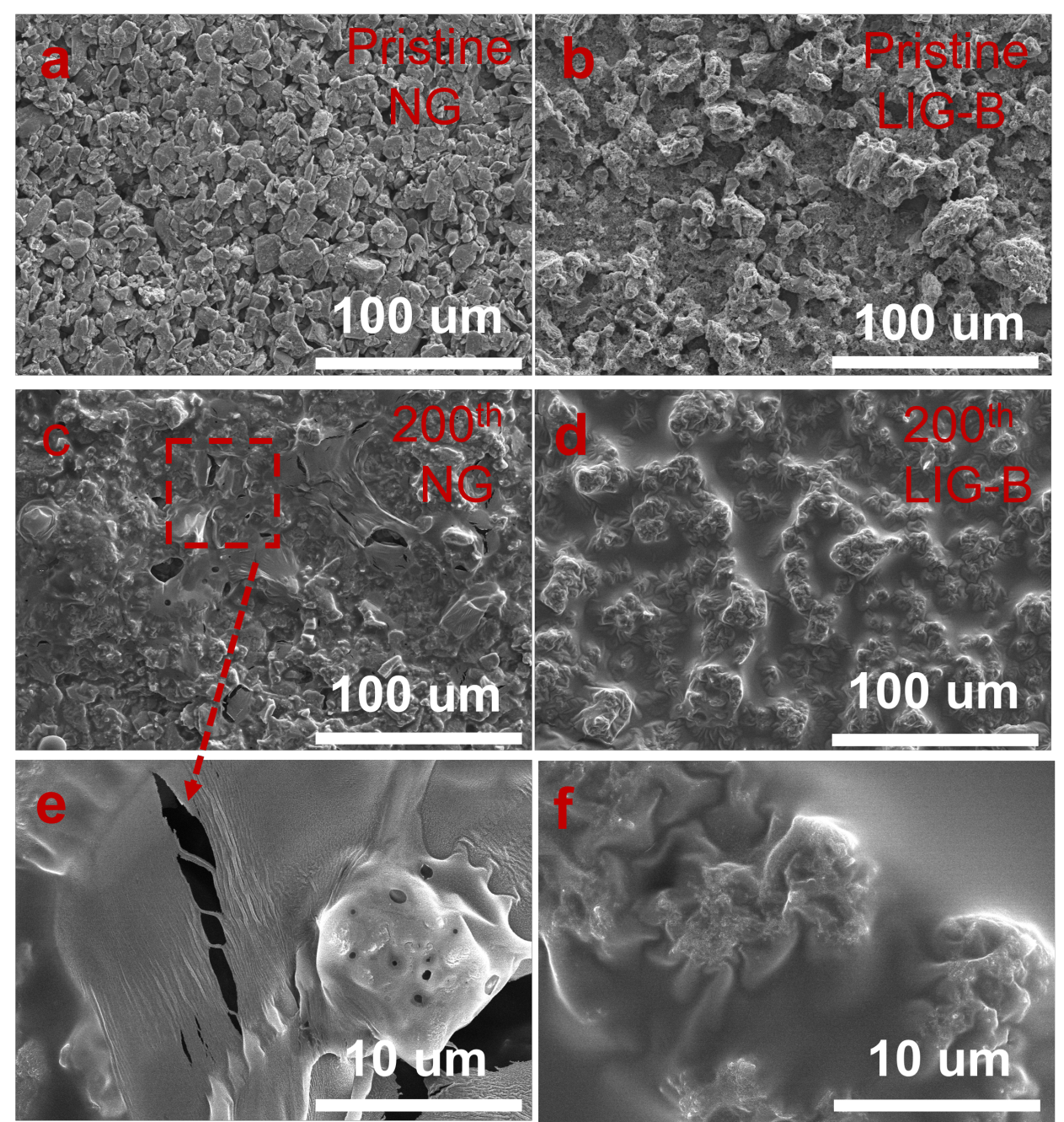


**Fig. S14.** (a-b) SEM images of pristine NG and LIG-B. (c-f) SEM images of NG and LIG-B after 200^th^ cycles.


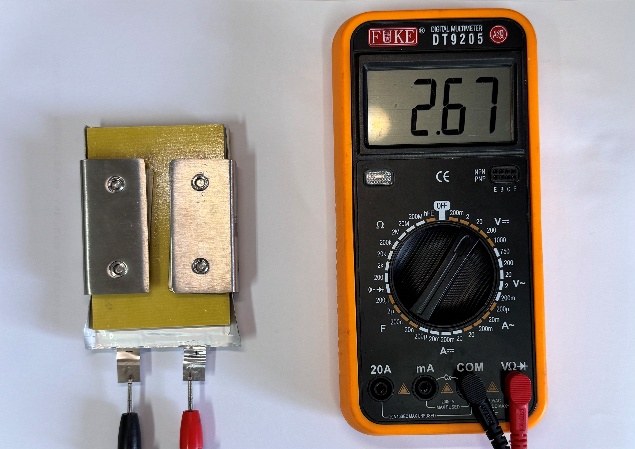


**Fig. S15.** The open-circuit voltage of LFP//LIG-B pouch cell


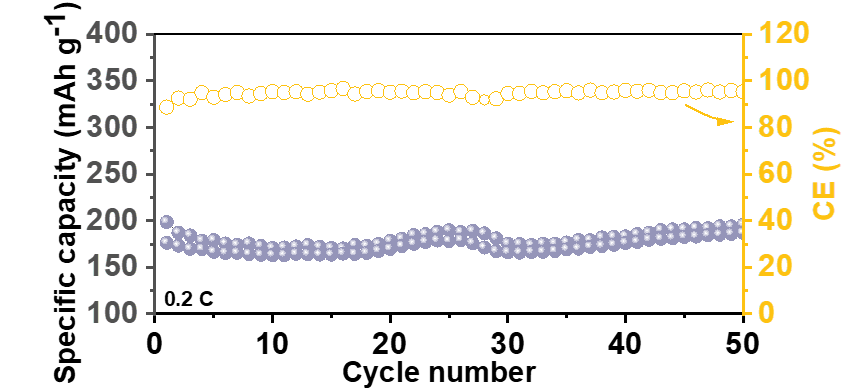


**Fig. S16**. Cycling performances of LFP//LIG-B pouch cell at current density of 0.2C


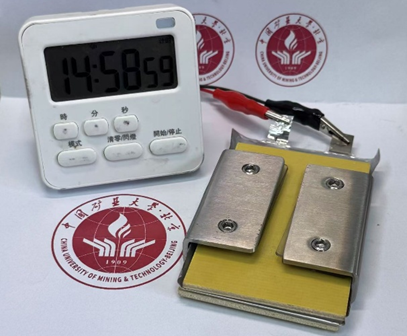


**Fig. S17**. The application demonstration of LFP//LIG-B pouch cell

**Table S1. Proximate and ultimate analyses of the coal.**

| Sample | Proximate analysis (wt%) | | | | | Ultimate analysis (wt%) | | | | | |
| --- | --- | --- | --- | --- | --- | --- | --- | --- | --- | --- | --- |
| coal of LIG-B | M_ad_ | A_d_ | V_daf_ | FC_d_ | C_daf_ | | H_daf_ | N_daf_ | O_daf_^*^ | S_t, daf_ |  |
|  | 0.90 | 31.79 | 14.94 | 58.02 | 89.53 | | 3.81 | 1.21 | 5.07 | 0.38 |  |

M_ad_, moisture; A_d_, ash yield; V_daf_, volatile matter; FC_d_, fixed carbon; C, carbon; H, hydrogen; N, nitrogen; O, oxygen; S_t_, total sulfur; ad, air-dry basis; d, dry basis; daf, dry and ash-free basis; *, by difference.

**Tabel S2 The proportion of C and O functional groups in the LIG-B XPS.**

| Simple | C 1s Area ratio | | | | | |
| --- | --- | --- | --- | --- | --- | --- |
| LIG-B | C-C  64.10% | | C-O  21.20% | | C**=**O  14.70% | |
|  | O 1s Area ratio | | | | | |
|  | C-O | -OH | | O-C**=**H | | C**=**O |
|  | 24.93% | 26.36% | | 27.74% | | 20.97% |

**Table S3 Performance comparison of the LIG-B anode with the previously reported common coal-based carbon anode materials.**

| **Coal-based carbon** | **Current density** | **Initial**  **Reversible Capacity**  **(mAh g^-1^)** | **Cycle performance** | **Rate capability**  **(mAh g^-1^)** | **Ref** |
| --- | --- | --- | --- | --- | --- |
| A-900 | 0.1 A/g | 384.5 | 82.9%  100 cycles | 150 at 2000 mA/g | [1] |
| BCG-2800 | 2 C | 324.6 | 95.3 %  100 cycles | 120 at 5 C | [2] |
| expanded graphite | 0.2 C | 351.6 | 79.1 %  300 cycles | 50 at 2 C | [3] |
| B-CNTs | 25 mA/g | 337.6 | -  100 cycles | 100 at 1260 mA/g | [4] |
| CG-2500 | 2 C | 369 | 80%  200 cycles | 160 at 3 C | [5] |
| Anthracite and semi-coke | 0.1 A/g | 389.8 | - | - | [6] |
| **LIG-B** | **0.1 A/g** | **400** | **95%**  **900 cycles** | **220 at 2000 mA/g** | **This work** |

Note: “-”no information given in the research.

**Reference**

[1] X. Liu, H. Tao, C. Tang, X. Yang, *Chemical Engineering Science* **2022**, 248.

[2] B. Xing, C. Zhang, Y. Cao, G. Huang, Q. Liu, C. Zhang, Z. Chen, G. Yi, L. Chen, J. Yu, *Fuel Processing Technology* **2018**, 172, 162.

[3] R. Deng, F. Chu, H. Yu, F. Kwofie, M. Qian, Y. Zhou, F. Wu, *Fuel Processing Technology* **2022**, 227.

[4] J. Li, Y. Cao, L. Wang, D. Jia, *Rsc Advances* **2017**, 7, 34770.

[5] M. Shi, C. Song, Z. Tai, K. Zou, Y. Duan, X. Dai, J. Sun, Y. Chen, Y. Liu, *Fuel* **2021**, 292.

[6] M. Zhong, J. Yan, H. Wu, W. Shen, J. Zhang, C. Yu, L. Li, Q. Hao, F. Gao, Y. Tian, Y. Huang, S. Guo, *Fuel Processing Technology* **2020**, 198.
